# Abnormal Brain Functional Network Dynamics in Acute CO Poisoning

**DOI:** 10.3389/fnins.2021.749887

**Published:** 2021-11-11

**Authors:** Hongyi Zheng, Hongkun Liu, Gengbiao Zhang, Jiayan Zhuang, Weijia Li, Wenbin Zheng

**Affiliations:** Department of Radiology, The Second Affiliated Hospital, Medical College of Shantou University, Shantou, China

**Keywords:** independent component analysis, adult, children, acute carbon monoxide poisoning, dynamic functional network connectivity (dFNC), fMRI

## Abstract

**Aims:** Carbon monoxide poisoning is a common condition that can cause severe neurological sequelae. Previous studies have revealed that functional connectivity in carbon monoxide poisoning is abnormal under the assumption that it is resting during scanning and have focused on studying delayed encephalopathy in carbon monoxide poisoning. However, studies of functional connectivity dynamics in the acute phase of carbon monoxide poisoning may provide a more insightful perspective for understanding the neural mechanisms underlying carbon monoxide poisoning. To our knowledge, this is the first study that explores abnormal brain network dynamics in the acute phase of carbon monoxide poisoning.

**Methods:** Combining the sliding window method and k-means algorithm, we identified four recurrent dynamic functional cognitive impairment states from resting-state functional magnetic resonance imaging data from 29 patients in the acute phase of carbon monoxide poisoning and 29 healthy controls. We calculated between-group differences in the temporal properties and intensity of dFC states, and we also performed subgroup analyses to separately explore the brain network dynamics characteristics of adult vs. child carbon monoxide poisoning groups. Finally, these differences were correlated with patients’ cognitive performance in the acute phase of carbon monoxide poisoning and coma duration.

**Results:** We identified four morphological patterns of brain functional network connectivity. During the acute phase of carbon monoxide poisoning, patients spent more time in State 2, which is characterized by positive correlation between SMN and CEN, and negative correlation between DMN and SMN. In addition, the fractional window and mean dwell time of State 2 were positively correlated with coma duration. The subgroup analysis results demonstrated that the acute phase of childhood carbon monoxide poisoning had greater dFNC time variability than adult carbon monoxide poisoning.

**Conclusion:** Our findings reveal that patients in the acute phase of carbon monoxide poisoning exhibit dynamic functional abnormalities. Furthermore, children have greater dFNC instability following carbon monoxide poisoning than adults. This advances our understanding of the pathophysiological mechanisms underlying acute carbon monoxide poisoning.

## Introduction

Carbon monoxide (CO) poisoning is a serious health hazard, with nearly half of all CO poisoning patients developing neurobehavioral or cognitive sequelae (Hopkins and Woon, 2006), manifesting as neurological disorders with sudden onset of psychosis and extrapyramidal or pyramidal system abnormalities (Kudo et al., 2014). The long course of the disease, poor prognosis, and high disability rate significantly impair the quality of life of patients and impose a heavy burden on society and families. Carbon monoxide poisoning is more like a systemic disease and has a different physiological mechanism from general cerebral hypoxia. Carbon monoxide (CO) has a strong affinity for hemoglobin-containing proteins. With an affinity for hemoglobin (Hb) that is 250 times greater than that of oxygen, CO reduces the oxygen-carrying and oxygen-releasing capacity of Hb by competitively binding Hb with oxygen and by increasing the structural stability of Hb. In addition, mitochondrial cytochrome c oxidase (COX; complex IV) has only a threefold preference for CO compared to O2. With COX inhibited, oxidative phosphorylation slows down, decreasing ATP production in tissues, such as the brain and heart. Other complexes in the electron transport chain continue to shuttle electrons, generating superoxide, leading to further damage of cells and tissues (Rose et al., 2017). General cerebral hypoxia causes brain cells to undergo anaerobic metabolism, resulting in reduced ATP and increased lactate levels. Brain cells have impaired transmembrane transport and sodium and water retention occurs. The cells release excitatory transmitters, loss of calcium ions, and ultimately activation of the apoptotic cascade of cell activation (Douglas-Escobar and Weiss, 2015). However, more studies have been conducted on delayed encephalopathy following acute CO poisoning (Hou et al., 2013; Lee et al., 2018; Wu et al., 2020a, b), in which the main pathological changes are extensive demyelination of the brain’s white matter, bilateral symmetrical pallidocyte ischemia, and necrosis. There are only a few studies on brain function alterations during the acute phase of CO poisoning (Dinghua et al., 2013).

Moreover, prior research was limited due to technological constraints and tended to ignore the temporal properties of brain activity, with most studies focusing on the functional spatial localization of the brain, yet the temporal properties are quite important. Numerous studies have recently demonstrated that brain is not static but is a dynamic and complex system that is constantly changing at microscopic time scales to adapt to its environment (Deco and Kringelbach, 2014; Preti et al., 2017). Dynamic methods, represented by the sliding time window approach, have been extensively employed for research in areas such as cognitive function, psychiatric disorders, and lifelong development. As dynamical methods advance, a growing interest in the temporal properties of the brain has increased, of which dynamic functional network connectivity (dFNC) is a promising research direction. As a new approach, dFNC has demonstrated its effectiveness in various diseases and conditions (Bhinge et al., 2019; Salman et al., 2019; Zhu et al., 2019). However, no systematic studies have been conducted to explore the dynamic FC characteristics of CO intoxication as a disorder with severe neurobehavioral or cognitive sequelae. Therefore, the objective of this study was to explore whether patients with CO poisoning present with abnormal dFNC characteristics during the acute phase and to determine whether these characteristics are correlated with clinical variables and coma duration.

## Materials and Methods

### Participants

The acute carbon monoxide poisoning group consisted of 31 patients treated for acute carbon monoxide poisoning in our hospital between December 2020 and March 2021. The diagnostic criteria (Jiang et al., 2020) were as follows: (1) contact history of exposure to higher CO inhalation; (2) symptoms and signs of acute central nervous system damage; and (3) timely determination of CO Ab in blood conformed to the national diagnostic criteria. There were 10 males and 21 females in the diseased group. The time interval of exposure to high-concentration CO during MRI examination was no more than 3 days. Although patients in the acute carbon monoxide poisoning group were treated with hyperbaric oxygen and electrolyte disturbance after admission, their clinical symptoms and signs were slightly reduced, but they did not meet the recovery criteria, and they still showed a mental and cognitive decline. We scored all subjects with MMSE and recorded their coma time and hemoglobin concentrations. The control group consisted of nine males and 20 females. There was no significant difference in age and sex between the two groups (*P* > 0.05). All the patients have signed the informed consent. This study was approved by the Ethics Committee of the Second Affiliated Hospital of Shantou University.

### Data Acquisition and Preprocessing

Structural MRI and resting-state functional MRI data were collected using a 3 T GE MR scanner. Resting-state data were acquired using single-shot gradient-echo EPI sequence (repetition time = 2,000 ms; echo time = 30 ms; flip angle = 90°; field of view = 240 × 240 mm^2^; matrix size = 64 × 64; number of slices = 25; and voxel size = 3.43 × 3.43 × 5.0 mm^3^ with no gap; and 210 volumes acquired in 7 min). Data preprocessing was performed using the Resting-State fMRI Data Analysis Toolbox plus V1.21 (RESTplus V1.21^1^). The preprocessing steps include: (1) removal of the first 10 time points; (2) slice timing; (3) realign. The dFC patterns were significantly affected by the head motion of subjects (Savva et al., 2020). Therefore, we imposed restrictions on the head motion parameters, and participants with excessive head motion (translation > 2.5 mm in any plane or rotation > 2.5° in any direction) at any time during the scan were excluded from further analysis. The head motion between the case group and the normal group was analyzed (Siegel et al., 2014), and there was no statistical difference between the two groups (Supplementary Table 2 provides details of the statistical results of head movement parameters); (4) normalize; (5) smoothed with a 6 mm full-width at half-maximum (FWHM) Gaussian kernel; (6) regressing out the nuisance variables [Friston’s 24 head motion parameters (Friston et al., 1996), white matter, and cerebrospinal fluid signals, and linear trend. More and more evidence shows that global signal contains valuable information (Liu et al., 2017; Xu et al., 2018), so we did not perform global signal regression].

### Group Independent Component Analysis

The preprocessed fMRI data were decomposed into functional networks with a group-level spatial independent component analysis (ICA) using GIFT^2^ (version 3.0a). To improve functional segmentation, we used a relatively high-order model (100 individual components) (Kiviniemi et al., 2009). Two data reduction steps (subject-specific and group level) were performed. We decomposed the grouped data into 100 independent components (ICs) using Infomax algorithm (Bell and Sejnowski, 1995) and repeated this step 100 times using ICASSO algorithm to evaluate the reproducibility or stability of ICs (Himberg et al., 2004). Finally, the ICs for each subject were derived from the group ICA back reconstruction step (Calhoun et al., 2001) and were converted into z-scores.

Based on the selection criteria and templates provided by previous studies (Allen et al., 2014; Kim et al., 2017), we ultimately selected 51 meaningful components. We divided the 51 ICs selected into seven functional networks: basal ganglion network (BG), default mode (DMN) network, cognitive executive network (CEN), sensorimotor network (SMN), auditory network (AUD), visual network (VIS), and cerebellar networks (CB). In order to remove the remaining noise source, the time process of the selected integrated circuit is post-processed. We applied the following post-processing steps to the time course of each IC, including linear, quadratic, and cubic de-trending; multiple regression of the movement parameters; despising detected outliers; and low-pass filtering with a high-frequency cutoff of 0.15 Hz.

### Dynamic Functional Connectivity Estimation

Dynamic functional connectivity analysis was examined using (FNC) toolbox in GIFT using two approaches: a sliding window approach and k-means clustering. By using the sliding window method, resting-state time series data were segmented into a 22-repetition time (TR) window with a size of 44 s, which is convolved with sigma 3-TR of Gaussian. The window was slid step-wise by 1 TR along the 200-TR length scan (400 s), resulting in 178 consecutive windows across the entire scan. Based on previous research, 44 S segment window length provides a good trade-off capability to better address the dynamic quality of functional connectivity and correlation matrix estimation (Allen et al., 2014). To promote sparsity in estimation, a penalty on the L1 norm was imposed in the graphic LASSO framework (Friedman et al., 2008). We explore FC patterns through the K-means clustering algorithm. We estimated the similarity between windowed FC matrices using the L1 distance (Manhattan distance) function. We repeated the k-means clustering analysis 100 times to increase the chance of escaping the local minima. Based on the elbow criterion (Allen et al., 2014), all windows of all subjects were clustered into four dFNC states.

We examine the temporal properties of dynamic functional connection states with three different variables: mean dwell time, fractional windows, and number of transitions. The mean dwell time represents how long the participant stayed in a certain state. The fractional window is the proportion of time spent in each state as measured by percentage. The number of transitions represents how many times either state changed from one to the other, counting the number of times a switch occurred.

### Statistical Analysis

Differences in demographic and clinical characteristics between groups were analyzed using the chi-square test for categorical data and Student’s *t*-test for normally distributed data. Because of the non-normality of the dynamic measures, the Mann–Whitney *U* test, a non-parametric test, was used in this study to detect the difference between carbon monoxide poisoning group and healthy controls. The statistical significance threshold was set at *p* < 0.05 and corrected for multiple comparisons using the FDR. We performed Spearman’s correlation analysis to investigate the relationship between the dFNC characteristics and the clinical variables in the carbon monoxide poisoning group. The results were considered significant at *p* < 0.05 uncorrected. In addition, we performed a subgroup analysis, in which the adult CO poisoning group (*n* = 13, >18 years of age) and the children CO poisoning group (*n* = 16, <18 years of age) were compared with the healthy control group. The results were considered significant at *p* < 0.05, FDR corrected.

## Results

### Clinical Results

No significant differences were observed between healthy control and CO poisoning groups in terms of gender and age (*p* = 0.787, two-sample *t*-test). The CO poisoning groups had lower MMSE scores than the healthy control group (*p* = 0.000, two-sample *t*-test). Detailed demographic and clinical information is displayed in Table 1.

**TABLE 1:** Participant demographic and clinical characteristics.

	Patients	Controls	*p*-value
Gender (male/female)	9/20	9/20	–
Age	20.89 (11.55)	20.03 (9.78)	0.760
MMSE	15.82 (5.86)	25.82 (4.18)	0.000*
Duration of coma (min)	101.33 (96.97)	–	
COHb (%)	33.46 (12.08)	–	

*MMSE, Simple Mental State Examination Scale; P-values were obtained by a two-sample t-test for age, MMSE, and chi-square test for gender difference.*

*P < 0.05.

### Intrinsic Connectivity Networks

**FIGURE 1:**
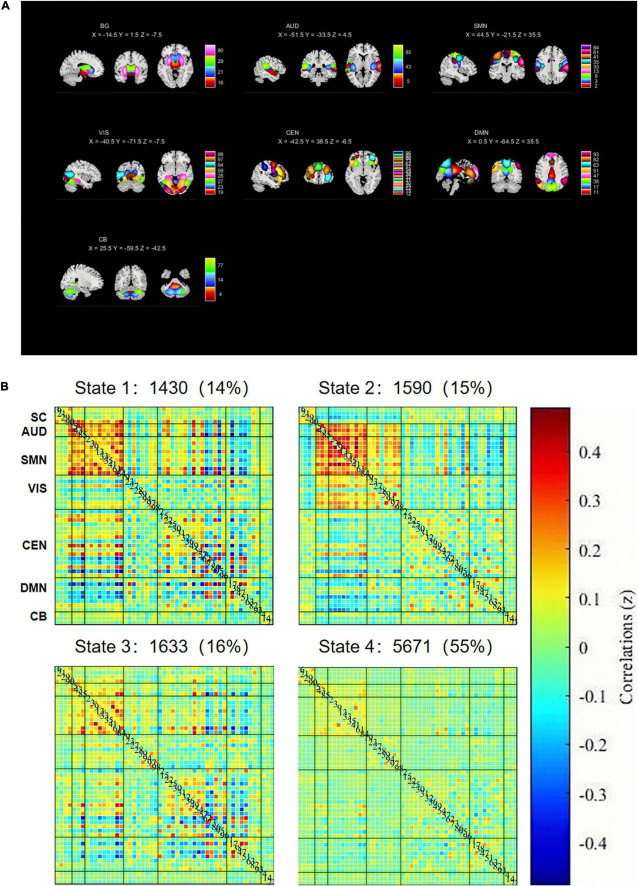
**(A)** Basal ganglia (BG), auditory (AUD), sensorimotor (SMN), visual (VIS), default mode (DMN), cognitive executive (CEN), and cerebellar networks (CB). **(B)** The centroid of each FNC state and its total number (percentage) of occurrence in each state.

According to their anatomical information and assumed functional characteristics, these 51 divided into the following seven networks (Figure 1A): BG (ICs 16, 21, 29, 90); AUD (ICs 5, 43, 85); SM (ICs 2, 3, 9, 13, 33, 35, 41, 61, 84); VIS (ICs 19, 23, 27, 28, 59, 94, 97, 98); CEN (ICs 12, 15, 22, 25, 30, 31, 32, 36, 49, 54, 57, 62, 73, 86, 95, 96); DMN (ICs 11, 17, 38, 47, 51, 63, 82, 93); and CB (IC 4, 14, 77). The distribution of these networks is basically the same as the previous research (Allen et al., 2014; Kim et al., 2017). The detailed component labels and peak coordinates of ICs are provided in the Supplementary Tables.

### Dynamic Functional Network Connectivity

The K-clustering was repeated 100 times to obtain dFC states, based on an optimal number of clusters of four according to elbow criteria (Allen et al., 2014), where each cluster represented one dFC state. Using k-means clustering approach, we identified four dFNC states (Wu et al., 2019; Figure 1B). State I (14% dFNC) was characterized by connections between SMN, CEN, and DMN networks with positive and negative couplings, and significant positive correlation within SMN and AUD networks. In State 2, which accounts for 15% of all windows, SMN was positively correlated with VIS and CEN, while DMN was negatively correlated with AUD, VIS, and SMN. State 3 (16% dFNC) is similar to State 1, but the connectivity within and between networks is weaker than that in State 1. In State 4, which accounts for 55% of all windows, FNC between all functional networks was very sparse.

Group differences in the temporal properties of dFNC states are displayed in Table 2. Compared to HCs, patients with acute CO poisoning had significantly increased fractional windows and mean dwell time in State 2 (for fraction, *p* = 0.000, FDR corrected; for mean dwell time, *p* = 0.000, FDR corrected).

**TABLE 2:** The results of Mann–Whitney *U* test.

	Patients (*n* = 29)	Controls (*n* = 29)	*p*-value
**Fraction (%)**			
State 1	8.98 (0.00–75.84)	1.12 (0.00–83.15)	0.728
State 2	12.92 (0.00–85.39)	0.00 (0.00–43.26)	0.03*
State 3	3.37 (0.00–92.70)	4.49 (0.00–93.82)	0.823
State 4	47.19 (0.00–100.00)	71.91 (1.69–100.00)	0.108
**Dwell time**			
State 1	13.66 (0.00–45.00)	2.00 (0.00–49.33)	0.34
State 2	15.00 (0.00–152.00)	0.00 (0.00–33.00)	0.05*
State 3	6.00 (0.00–82.50)	8.00 (0.00–83.50)	0.386
State 4	26.00 (0.00–178.00)	46.00 (3.00–178.00)	0.34
Transition	6.00 (0.00–14.00)	4.00 (0.00–10.00)	0.19

*According to the type and distribution of the data, fractional windows, transition number, and mean dwell time are expressed as median (IQR). Mann–Whitney *U* test for two groups.*

Asterisk (*) means p < 0.05, FDR corrected.

### Dynamic FC States and Clinical Symptoms

**FIGURE 2:**
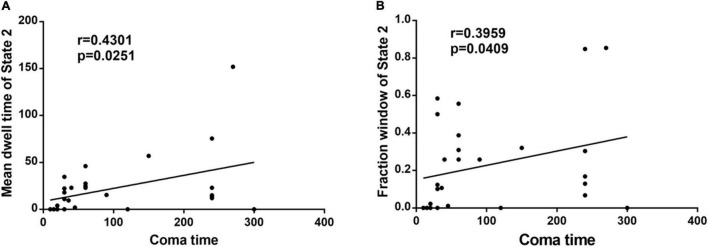
**(A)** The mean dwell time of State 2 is positively associated with coma time in acute CO poisoning patients. **(B)** The fraction window of State 2 is positively correlated with coma time in acute CO poisoning patients.

In the acute CO poisoning group, we found a positive association between the mean dwell time of State 2 and coma time (Figure 2A). In addition, the fraction window of State 2 was positively correlated with coma time (Figure 2B).

### Subgroup Analysis

The subgroup analysis results revealed that State 2 of children patients had significantly higher fraction and mean dwell time than HC group (for fraction, *p* = 0.000, FDR corrected; for mean dwell time, *p* = 0.000, FDR corrected). However, State 4 of children patients had a significantly lower fraction and mean dwell time than HC group (for fraction, *p* = 0.01, FDR corrected; for mean dwell time, *p* = 0.034, FDR corrected), and children patients with acute CO poisoning make more transitions than HCs (*p* = 0.006, FDR corrected). No significant outcome was observed in adults with acute CO poisoning in all states compared with HC.

## Discussion

Dynamic functional connectivity has been widely used to explore brain function and thus may serve as a novel physiological biomarker of disease (Hutchison et al., 2013). The present study analyzed dFNC in patients with acute CO poisoning, including the temporal properties of FNC states and their relationship with coma duration and clinical cognitive measurement of patients. Patients with acute CO poisoning spent more time in State 2 than controls. Moreover, the temporal properties of State 2 were positively correlated with coma time in the patient group. Other clinical indicators did not correlate significantly with temporal characteristics of dFNC. This could be explained by the insignificant effect of CO Hb as an indicator of the clinical status of CO poisoning (Hampson and Hauff, 2008). In addition, the MMSE focuses primarily on direction and language function, has a low false negative rate in assessing moderate to severe cognitive impairment, and is susceptible to language and education levels.

In fact, the acute phase of carbon monoxide poisoning leads to demyelination of brain white matter (Park et al., 2014), and damage to brain white matter inevitably disrupts connections between various large brain regions, resulting in reduced information transmission efficiency of the brain functional network in patients with acute carbon monoxide poisoning. This may be the main reason for clinical symptoms, such as slowed reactions, memory loss, and reduced logical thinking ability in patients with carbon monoxide poisoning early in life. Our results found that patients with acute CO poisoning spend more time on State 2, with positive and negative coupling between SMN and CEN, and negative correlations between DMN and SMN, CEN. A communication between CEN and SMN networks (while inactivating the DMN) has been suggested to be necessary for translating effective cognitive processing into action (Sridharan et al., 2008). In addition, in State 2, there was a negative correlation between DMN and other functional networks (SMN, VIS, and CEN), and collaboration between the two networks, CEN and DMN, facilitated memory (Blumenfeld and Ranganath, 2006; Huijbers et al., 2011). Therefore, it is reasonable to speculate that state 2 is a compensatory state for patients in the acute phase of co poisoning. The increased frequency of expression of these functional couplings in patients with acute CO poisoning due to the reduced efficiency of information transfer in the functional brain network can be explained by a potential compensatory mechanism of the intrinsic brain network, leading to stronger synchronization. More convincingly, CO poisoning patients (19 individuals) showed State 2, whereas only nine normal individuals demonstrated State 2. In addition, we found a positive correlation between the fractional window and mean dwell time of State 2 and coma duration in patients. Thus, we hypothesized that the duration of coma after co poisoning affected the length of compensatory state. Based on these results, we defined State 2 as an abnormal compensatory state in patients with acute CO poisoning. The most frequently reoccurring State 4 has the weak connectivity patterns that resembled the static FNC patterns. In previous dFNC studies, a dynamic state that resembles the stationary FNC patterns typically accounts for the largest percentage of windows. This weakly connected state is more common in many psychiatric disorders, such as bipolar disorder (Rashid et al., 2014), schizophrenia (Damaraju et al., 2014; Fu et al., 2018), autism (Fu et al., 2019), and Parkinson (Kim et al., 2017). However, the patients with acute carbon monoxide poisoning do not exhibit such negative patterns in the occurrence of State 4. This may be related to the fact that carbon monoxide poisoning has a different pathophysiological mechanism than other psychiatric disorders. There is similar connection mode between state 1 and state 3, but the connection strength of state 1 is obviously stronger than that of state 3. We do not yet know the physiological significance of these two states, and statistically there were no inter-group differences in the temporal characteristics and intensity of the two states. Therefore, further detailed studies are needed.

The subgroup analysis results revealed that children with CO poisoning exhibited greater dFNC instability, including an increased incidence of State 2, a decreased incidence of State 4, and more frequent transitions between states. In contrast, no significant results were observed in the adult subgroup. Therefore, this compensatory state 2 is more likely to occur after carbon monoxide poisoning in children. In addition, children with acute carbon monoxide poisoning spent less time in state 4. Recent studies (Marusak et al., 2017) further suggest that this state may be associated with self-referential processing. We speculate that the reasons for this are that the first reason is that different levels of brain development, high levels of attention, and cognitive control processes develop during childhood and adolescence, and the emergence of these complex processes is supported by the reconfiguration and refinement of functional brain networks (Bunge and Wright, 2007; Luna et al., 2010). The second reason may be the existence of different collateral circulation compensations in the brain. Children and adults do have different collateral circulation compensations under certain brain diseases (Liu et al., 2020), but further detailed studies are needed to evaluate this hypothesis. Dynamic functional connectivity changes with age. Therefore, the results of this study indicate that children with carbon monoxide poisoning exhibit greater dFC instability than adults. It is not necessarily all carbon monoxide poisoning, but it could be age-related (Tian et al., 2018), so our conclusions should be treated with caution.

Certain limitations should be considered when interpreting our findings. First, most of our patients underwent varying degrees of hyperbaric oxygen therapy before their MRI scans. We cannot exclude the effect of hyperbaric oxygen treatment on our resting-state data. In future studies, it will be important to separate the abnormalities associated with CO poisoning from the associated effects of hyperbaric oxygen therapy on dynamic functional connectivity properties by comparing patients treated with hyperbaric oxygen therapy to those who did not undergo hyperbaric oxygen therapy. Second, considering the clinical simplicity, clinicians used the more generally applicable MMSE score for emergency patients with CO poisoning. In contrast, while MoCA scores are more extensive and more comprehensive in their assessment, especially in terms of space/execution, they are more cumbersome in their procedures and less convenient in clinical examination. It would be beneficial to further optimize the relevant operations and apply them to routine screening after CO poisoning. Third, when it comes to dFNC analysis, while the limited scan time in this study (i.e., 7 min) may be insufficient to investigate all aspects of dFNC changes. It has been shown that short scans can successfully capture different connection states, for example, 5 min. Longer scan times (>10 min) have been shown to better characterize the dynamic patterns of FC (Hindriks et al., 2016), and thus future work will extend the scan time further. Second, dFNC analysis lacks the gold standard for selecting dFNC estimation window size. Most dynamic functional connectivity studies estimate FC dynamics using a window size of 22 TRs (Kim et al., 2017; Wang et al., 2019). Therefore, it is reasonable to choose a window width of 22 TRs in this study.

## Conclusion

Using resting-state functional MRI data, GICA and sliding time window techniques can identify four FC states of time-varying functional network connectivity in the acute phase of carbon monoxide poisoning. Of these, State 2 is a compensatory state following carbon monoxide poisoning that occurs more frequently in pediatric patients and correlates with coma duration, inferring that dFNC analysis could serve as a potential imaging biomarker while also providing new insights into understanding the pathophysiological mechanisms underlying carbon monoxide poisoning.

## Data Availability Statement

The original contributions presented in the study are included in the article/Supplementary Material, further inquiries can be directed to the corresponding author/s.

## Ethics Statement

The studies involving human participants were reviewed and approved by the Second Affiliated Hospital, Medical College of Shantou University. Written informed consent to participate in this study was provided by the participants’ legal guardian/next of kin.

## Author Contributions

WZ, HL, and HZ contributed to conception and design of the study. HL and HZ wrote the first draft of the manuscript and completed the dynamic functional connection analysis of data and visualization of experimental results. WZ contributed to manuscript revision, read, and approved the submitted version. JZ, WL, and GZ completed data collation and statistical analysis. All authors contributed to the article and approved the submitted version.

## Conflict of Interest

The authors declare that the research was conducted in the absence of any commercial or financial relationships that could be construed as a potential conflict of interest.

## Publisher’s Note

All claims expressed in this article are solely those of the authors and do not necessarily represent those of their affiliated organizations, or those of the publisher, the editors and the reviewers. Any product that may be evaluated in this article, or claim that may be made by its manufacturer, is not guaranteed or endorsed by the publisher.

## Footnotes

1
http://www.restfmri.net/forum/REST

2
http://icatb.sourceforge.net/
