# Supplementary material for: Abnormal Brain Functional Network Dynamics in Acute CO Poisoning
Source: Front Neurosci. 2021 Nov 11;15:749887. doi: 10.3389/fnins.2021.749887 (PMC8636030; doi:10.3389/fnins.2021.749887)
Supplement: Supplementary file 1 [file Data_Sheet_1.docx]

**Supplementary Materials**

**Table S1. Peak activations and coordinates of the ICN spatial maps.**

| **ICNs** | **X** | **Y** | **Z** |
| --- | --- | --- | --- |
| **Basal ganglia network （4）** | | | |
| Caudate (21) | 12 | 17 | -1 |
| Subthalamus/hypothalamus (16) | 3 | -13 | -16 |
| Putamen (90) | -24 | 2 | -4 |
| Thalamus (29) | -9 | -15 | 10 |
| **Auditory domain (AUD)（3）** | | | |
| Superior temporal gyrus ([STG], 5) | 63 | -19 | -1 |
| Superior temporal gyrus ([STG], 43) | -39 | -25 | 8 |
| Superior temporal gyrus ([STG], 85) | -54 | -37 | 14 |
| **Sensorimotor domain (SM)（9）** | | | |
| Postcentral gyrus ([PoCG], 3) | -54 | -13 | 32 |
| Left postcentral gyrus ([L PoCG], 41) | -36 | -31 | 59 |
| Paracentral lobule ([ParaCL], 2) | -6 | -37 | 65 |
| Right postcentral gyrus ([R PoCG], 9) | 42 | -28 | 62 |
| Superior parietal lobule ([SPL], 13) | -18 | -58 | 65 |
| Supplementary motor area ([SMA], 84) | 12 | -4 | 53 |
| Postcentral gyrus ([PoCG], 33) | -21 | -34 | 59 |
| Postcentral gyrus ([PoCG], 35) | -36 | -19 | 44 |
| Supramarginal gyrus ([SMG], 61) | 63 | -25 | 32 |
| **Visual domain (VS)（8）** | | | |
| Calcarine gyrus ([CalcarineG], 19) | -6 | -97 | -4 |
| Middle occipital gyrus ([MOG], 94) | -51 | -76 | 8 |
| Middle occipital gyrus ([MOG], 98) | -27 | -94 | -1 |
| Cuneus (97) | -3 | -88 | 17 |
| Calcarine gyrus ([CalcarineG], 23) | -18 | -64 | 11 |
| Fusiform gyrus (28) | 33 | -40 | -10 |
| Inferior occipital gyrus ([IOG], 27) | 45 | -64 | -13 |
| Lingual gyrus ([LingualG], 59) | -21 | -67 | -7 |
| **Cognitive-control domain (CC)（16）** | | | |
| Left Inferior parietal lobule/Postcentral gyrus(L [IPL/PoCG], 96) | -57 | -28 | 35 |
| aInsula (22) | -30 | 23 | -1 |
| Inferior frontal gyrus ([IFG], 30) | -51 | 23 | 5 |
| Right inferior frontal gyrus ([R IFG], 36) | 45 | 17 | 26 |
| Left inferior frontal gyrus ([R IFG], 54) | -39 | 17 | 26 |
| Middle frontal gyrus ([MiFG], 12) | -27 | 53 | 8 |
| Left Supplementary motor area/Precentral gyrus (L [SMA/PreCG], 73) | -6 | 2 | 65 |
| Middle frontal gyrus ([MiFG], 32) | -33 | 41 | 32 |
| Supplementary motor area([SMA], 86) | -6 | 11 | 50 |
| Superior medial frontal gyrus ([SMFG], 62) | 0 | 41 | 38 |
| Precentral gyrus ([L PreCG], 57) | 54 | 5 | 20 |
| Inferior frontal gyrus ([IFG], 31) | 36 | 44 | -7 |
| ParaHippocampal gyrus ([PHG], 15) | -24 | -10 | -22 |
| pInsula (49) | 36 | 8 | 11 |
| Superior frontal gyrus ([SFG], 95) | -18 | 17 | 53 |
| Superior frontal gyrus ([SFG], 25) | -18 | -1 | 56 |
| **Default-mode domain (DM)（8）** | | | |
| Precuneus (63) | 0 | -67 | 56 |
| Precuneus + Cuneus (38) | -6 | -82 | 35 |
| Anterior cingulate cortex ([ACC], 47) | -3 | 56 | 14 |
| Posterior cingulate cortex ([PCC], 11) | -3 | -31 | 23 |
| Anterior cingulate cortex ([ACC], 82) | 6 | 35 | 14 |
| Posterior cingulate cortex ([PCC], 17) | -3 | -64 | 26 |
| Angular gyrus ([AG], 93) | -51 | -50 | 43 |
| Left Angular gyrus ([L AG], 51) | -51 | -64 | 41 |
| **Cerebellar domain (CB)（3）** | | | |
| Cerebellum ([CB], 4) | -12 | -64 | -43 |
| Cerebellum ([CB], 14) | -27 | -73 | -34 |
| Cerebellum ([CB], 77) |  |  |  |

**Basal ganglia network**

**
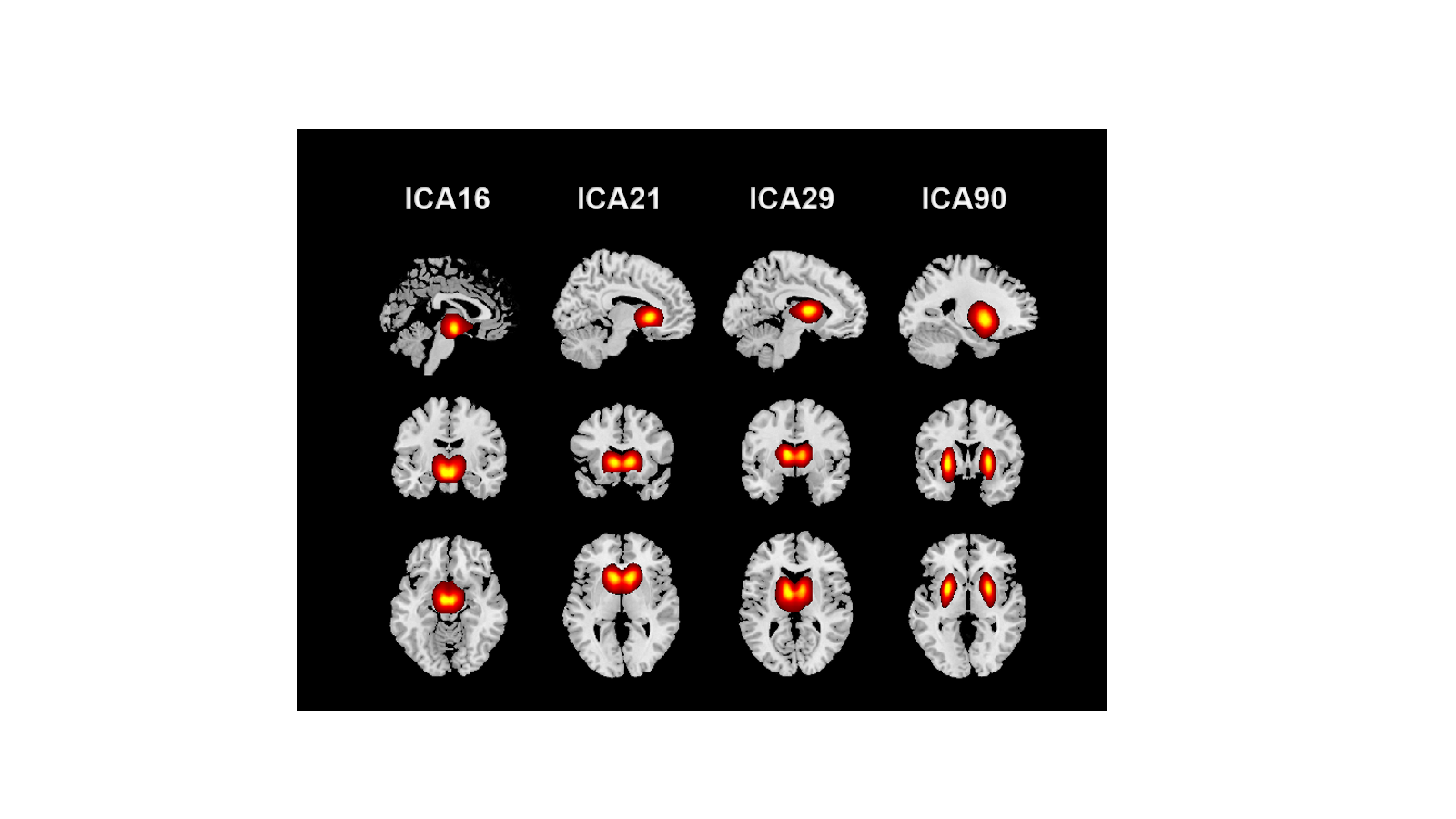
**

**Auditory network**

**
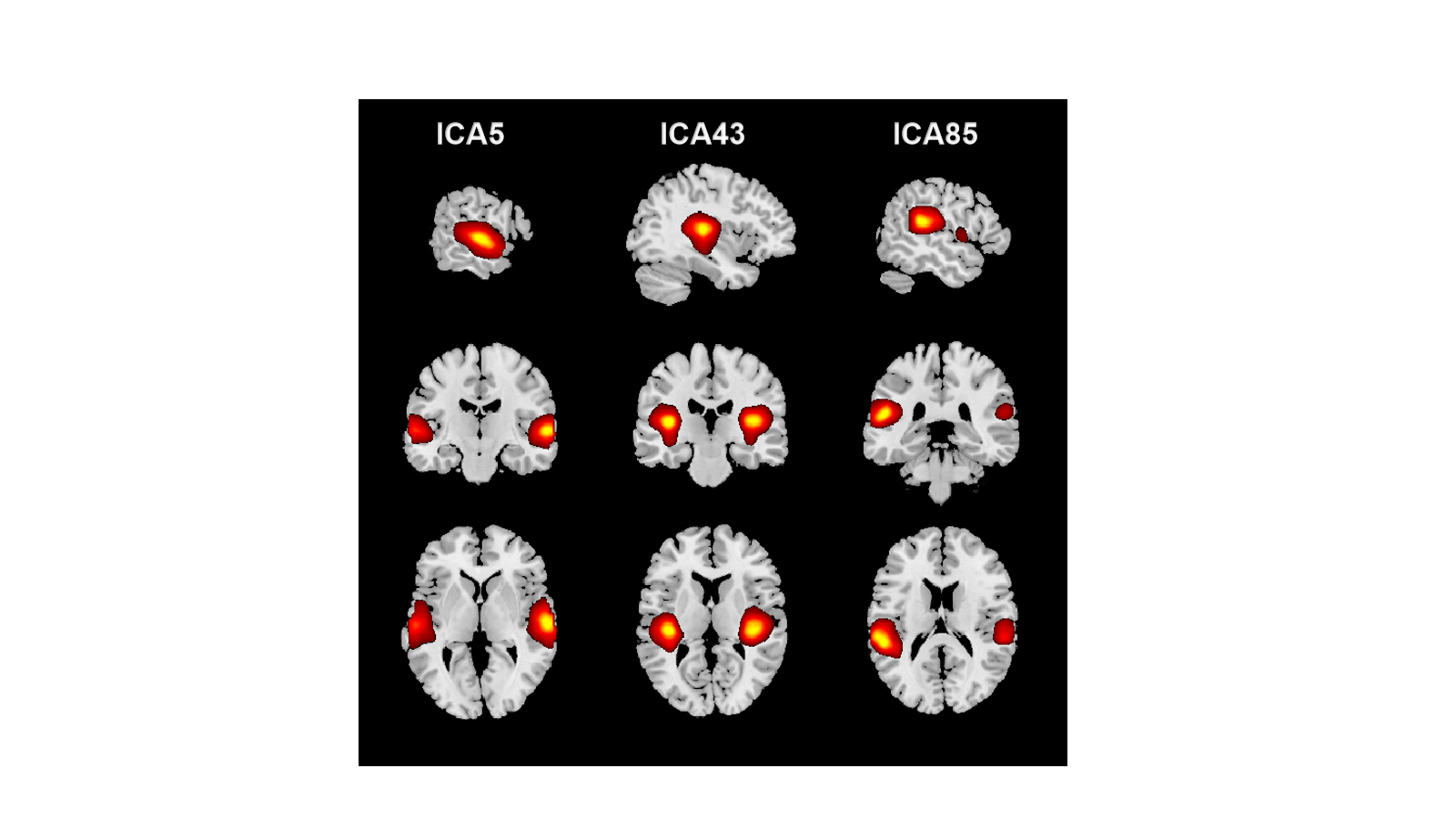
**

**Sensorimotor network**

**
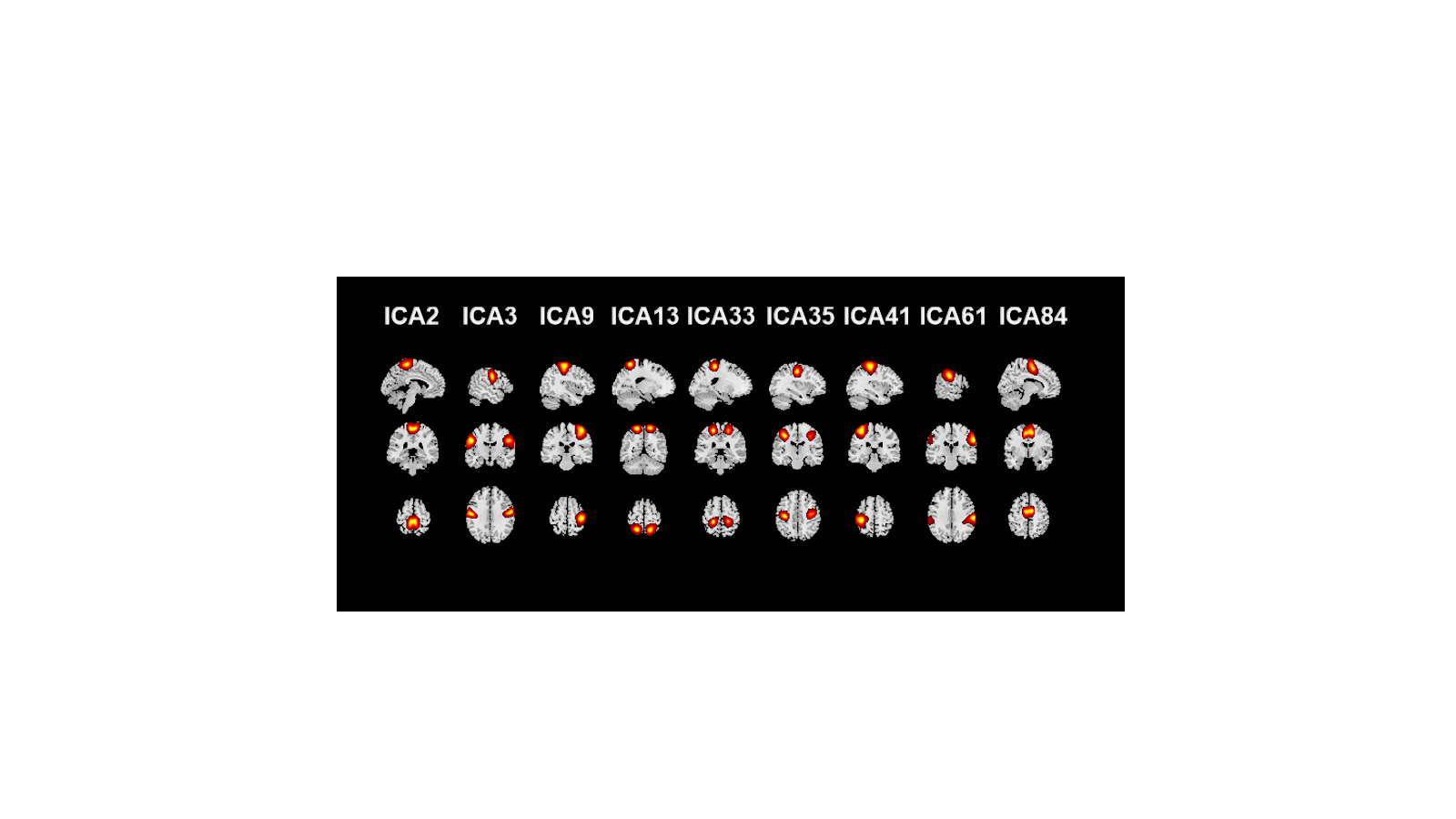
**

**Visual network**

**
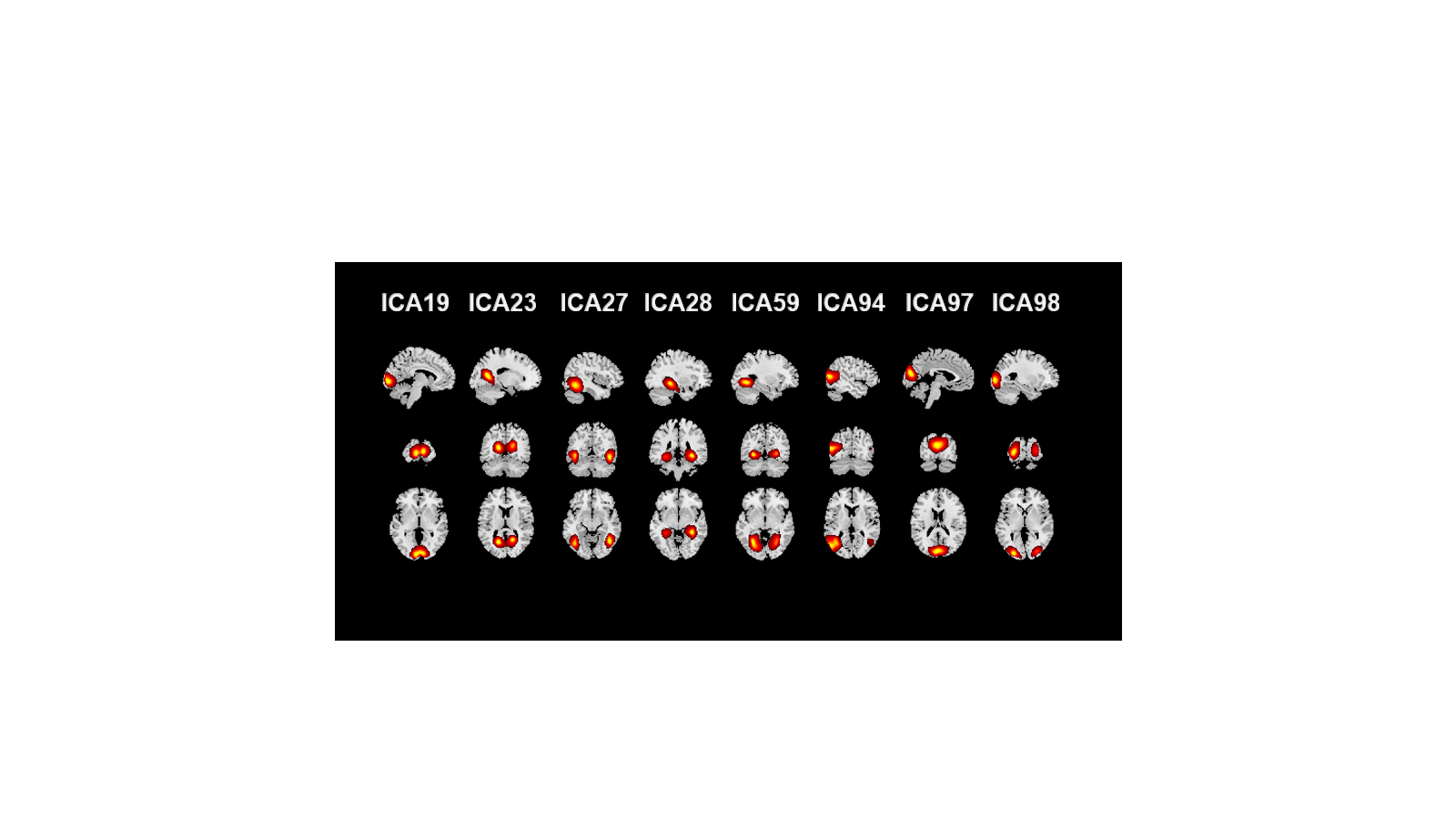
**

**Cognitive executive network**


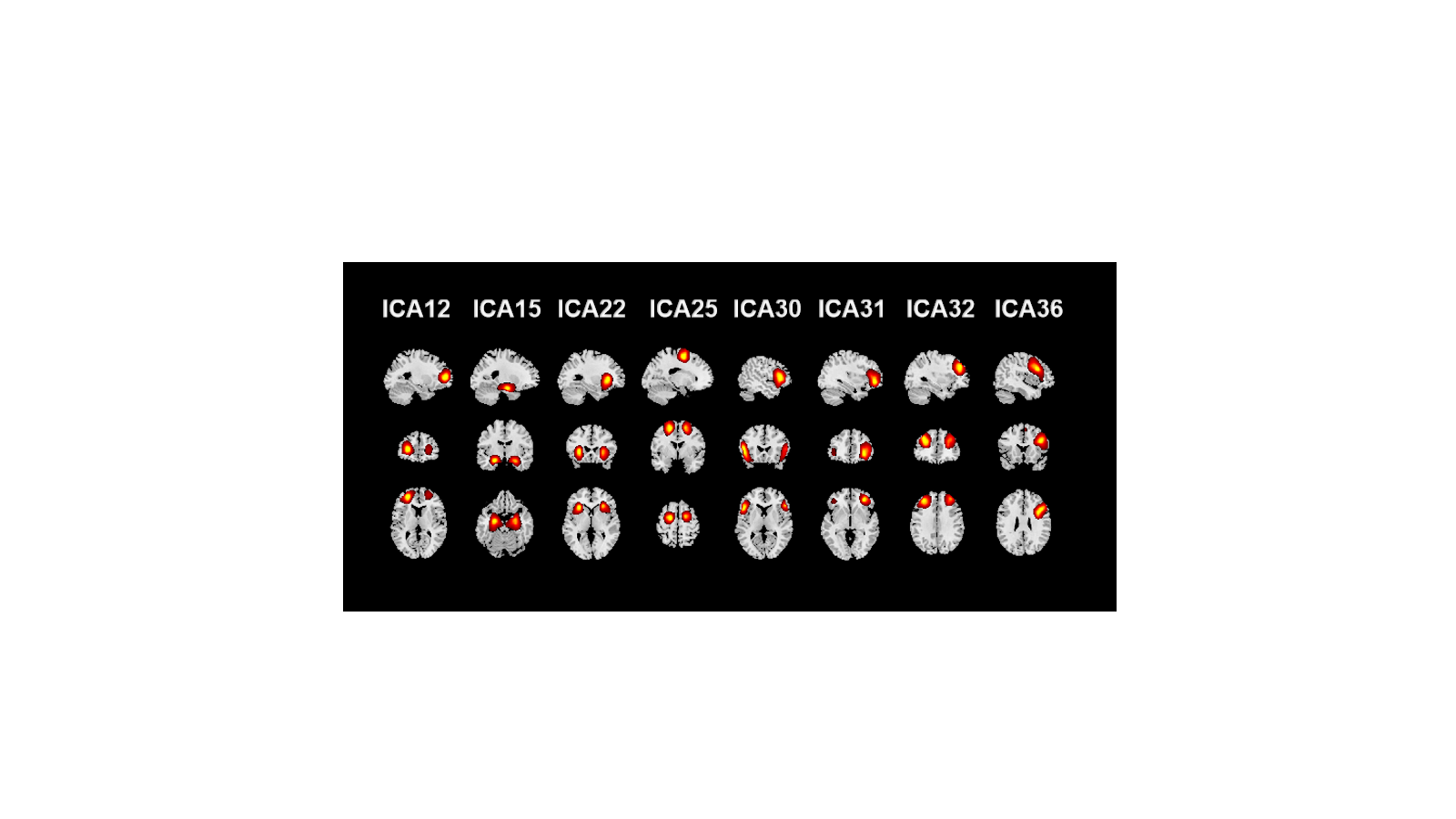


**
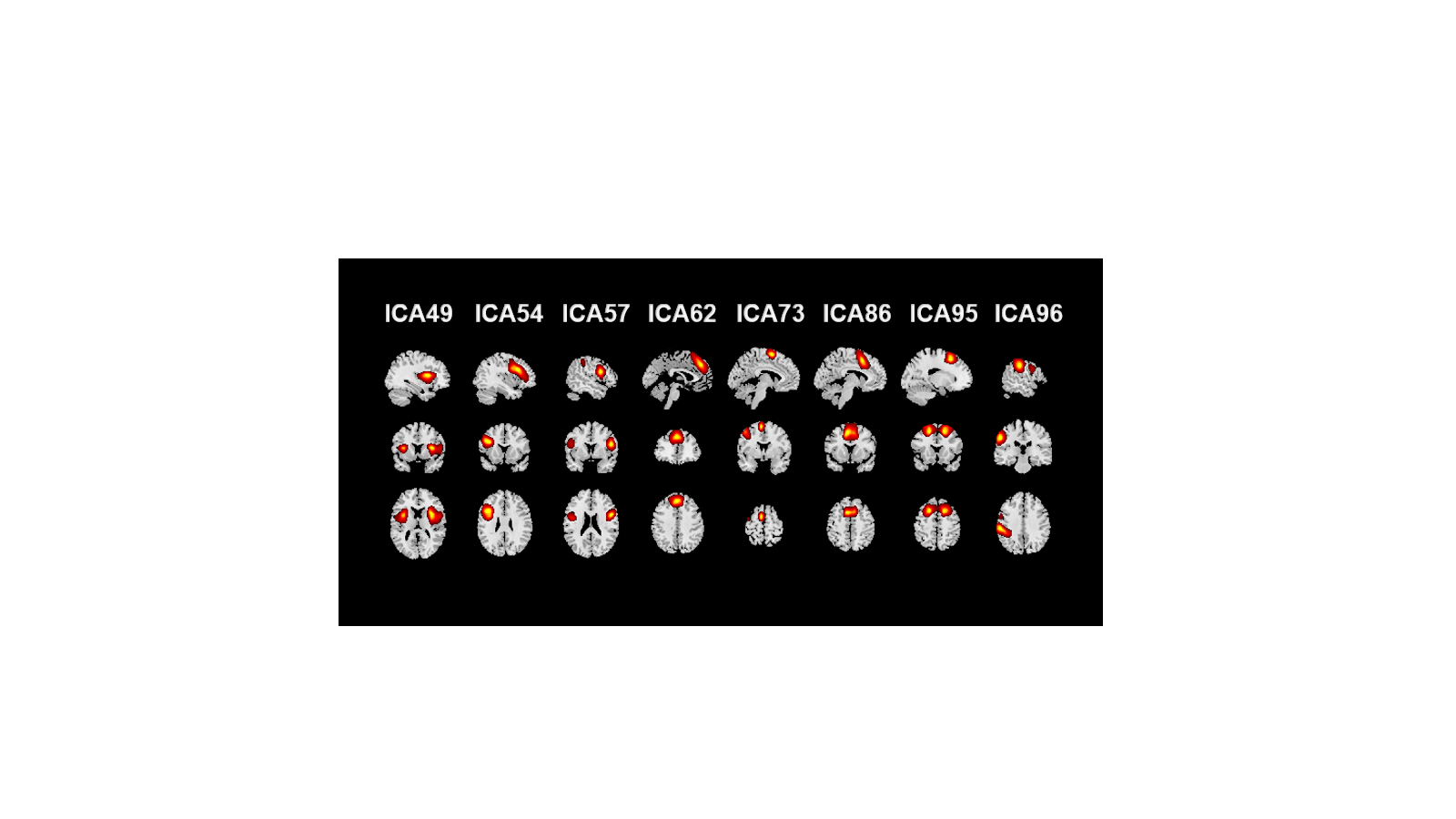
**

**Default mode network**

**
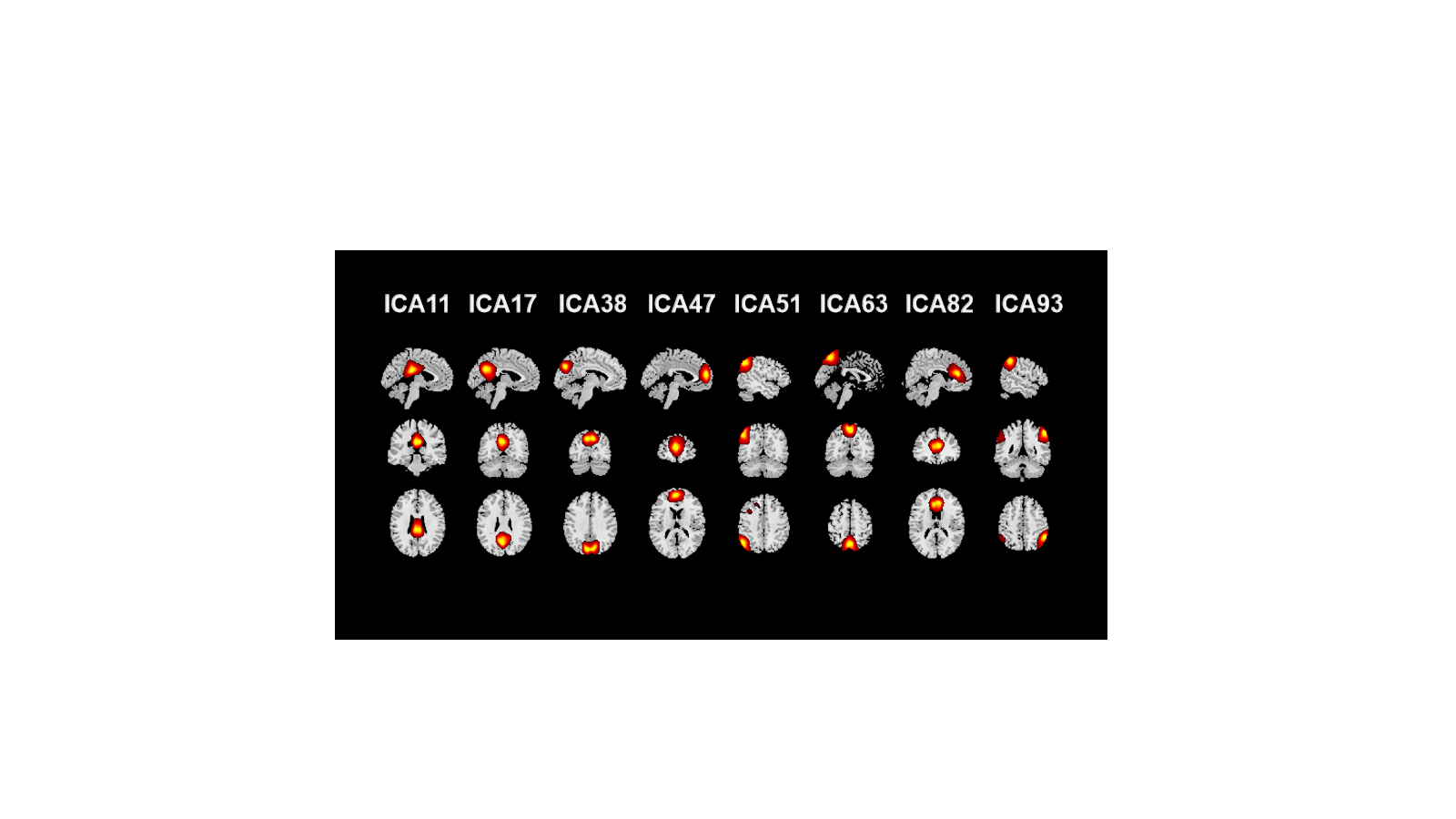
**

**Cerebellar network**

**
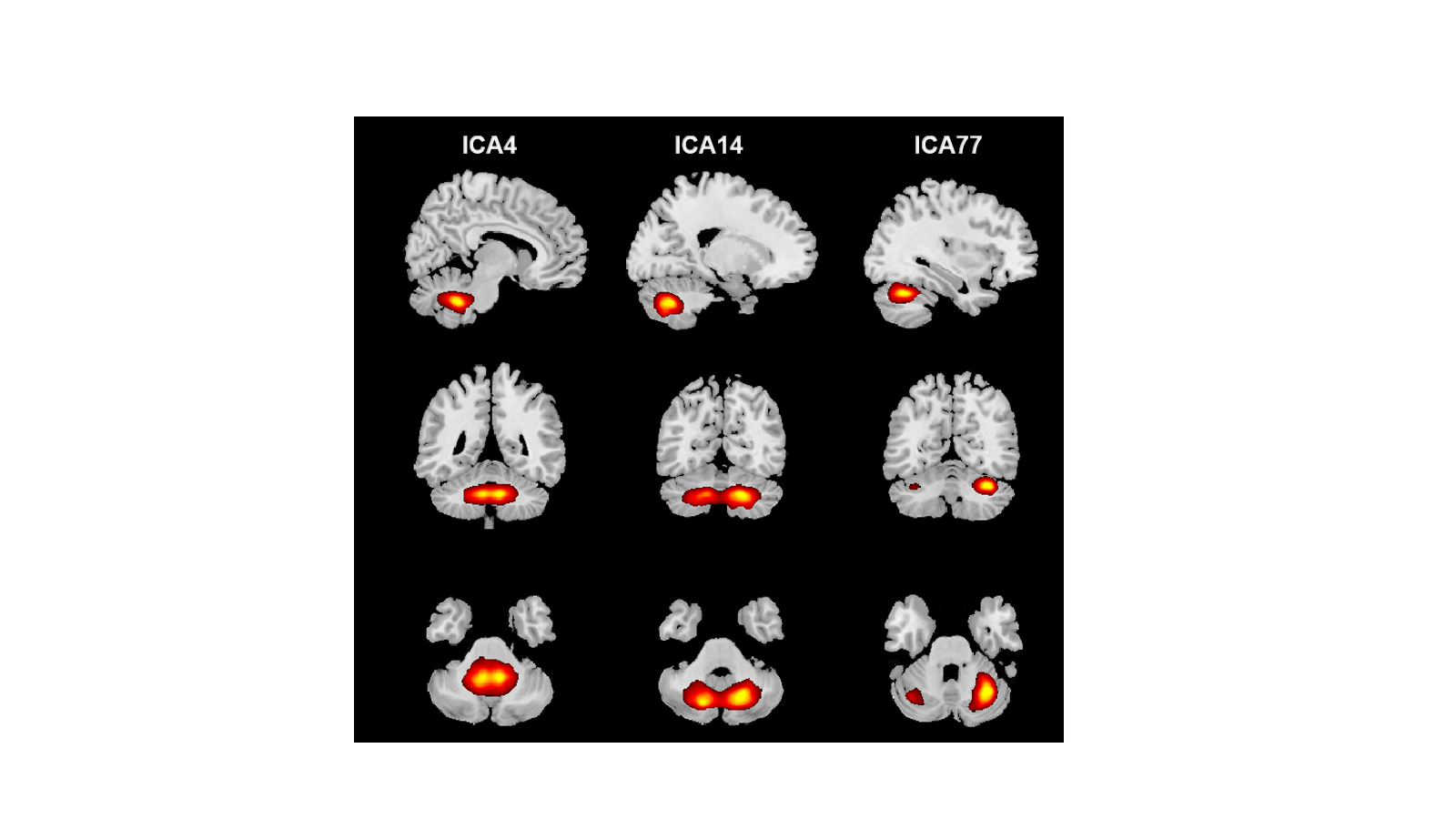
**

**Table S2. The head motion between the case group and the normal group**


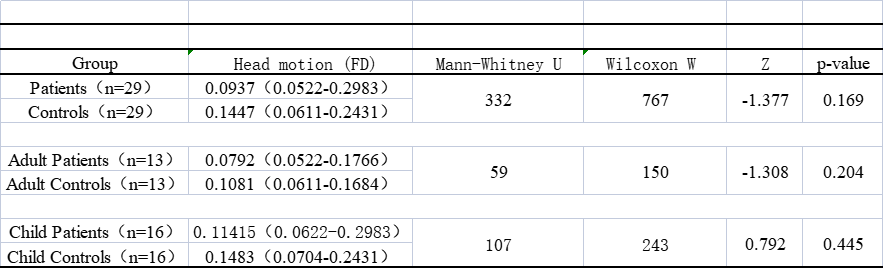


Continuous variables were expressed as median and interquartile range. Continuous parameters were checked for the normality of distribution using the Shapiro–Wilk test and compared using Mann-Whitney U test. A value of P<0.05 was considered as statistically significant.
